# In Vitro and In Vivo Protective Effects of Agaro-Oligosaccharides against Hydrogen Peroxide-Stimulated Oxidative Stress

**DOI:** 10.3390/polym15071612

**Published:** 2023-03-23

**Authors:** Lei Wang, Xiaoting Fu, Jimin Hyun, Jiachao Xu, Xin Gao, You-Jin Jeon

**Affiliations:** 1 College of Food Science and Engineering, Ocean University of China, Qingdao 266003, China; 2 Department of Marine Life Sciences, Jeju National University, Jeju 63243, Republic of Korea; 3 Marine Science Institute, Jeju National University, Jeju 63333, Republic of Korea

**Keywords:** agaro-oligosaccharides, ROS, oxidative stress

## Abstract

In our previous research, we investigated the anti-inflammatory activity of the agaro-oligosaccharides prepared from the agar of *Gracilaria lemaneiformis* (AO). In the present study, in order to further explore the bioactivities of AO, the antioxidant activity of AO was investigated in vitro in Vero cells and in vivo in zebrafish. AO scavenged alkyl, 1,1-diphenyl-2-picrylhydrazyl, and hydroxyl radicals at the IC_50_ value of 4.86 ± 0.13, 3.02 ± 0.44, and 1.33 ± 0.05 mg/mL, respectively. AO significantly suppressed hydrogen peroxide (H_2_O_2_)-stimulated oxidative damage by improving cell viability. This happened via suppressing apoptosis by scavenging intracellular reactive oxygen species (ROS). Furthermore, the in vivo results displayed that AO protected zebrafish against H_2_O_2_-stimulated oxidative damage by reducing the levels of intracellular ROS, cell death, and lipid peroxidation in a dose-dependent manner. These results indicate that AO effectively alleviated in vitro and in vivo oxidative damage stimulated by H_2_O_2_, and suggest the potential of AO in the cosmetic and functional food industries.

## 1. Introduction

Oxidative stress is caused by an imbalance between the generation and scavenging of reactive oxygen species (ROS) such as hydrogen peroxide (H_2_O_2_), anion radical, and hydroxyl radical [1]. H_2_O_2_, especially, can easily penetrate cells’ membranes and react with intracellular ions, which leads to cellular damage. Under the physiological conditions, a balance between ROS generation and scavenging occurs. In this case, ROS regulate various cellular functions such as differentiation and quiescence [1,2]. However, overproduction of ROS leads to cellular macromolecules damage such as proteins, DNA, and lipids, and subsequent disruption of cell functions [3]. The accumulation of cell dysfunction leads to various diseases such as aging, diabetes, cardiovascular diseases, inflammation, obesity, and cancer [4]. Therefore, the supplement of antioxidants to scavenge the excess ROS may be an ideal strategy to prevent diseases caused by oxidative stress.

Natural products possess a lot of advantages, such as high effectivity and low adverse effects. Natural products such as sugars, proteins, lipids, and vitamins from terrestrial or aquatic resources (including microorganisms, plants, and animals) have been used as medicinal material to treat diseases for a long time [5,6,7,8]. Natural products from seaweeds, such as pigments, polysaccharides, and lipids, possess various bioactivities including anti-diabetes, anti-cancer, antioxidant, anti-virus, anti-inflammatory, and anti-obesity effects [9,10]. In particular, seaweeds are rich in carbohydrates, and the polysaccharides and oligosaccharides isolated from seaweeds possess strong antioxidant activities [11,12,13].

Oligosaccharides isolated from natural resources have been widely used in medical, food, and cosmetic industries. Oligosaccharides possess a variety of health benefits such as antioxidant, anti-tumor, anti-coagulant, probiotic, and anti-inflammatory effects [14,15,16,17,18,19]. Zhao et al. investigated the antioxidant effect of oligosaccharides from mountain-cultivated ginseng and cultivated ginseng. The results indicate that the antioxidant activity of total oligosaccharides from mountain-cultivated ginseng displayed stronger antioxidant activity than that of cultivated ginseng [15]. Xiong et al. isolated the oligosaccharides from *Evodia lepta* by microwave-assisted extraction (MEO) and evaluated the antitumor activity of MEO. The results show that MEO effectively inhibited tumor cell growth [17]. Kim et al. isolated the oligosaccharides from *Leuconostoc lactis* SBC001 (LLO) and investigated the prebiotic and anti-inflammatory effects of LLO. The results show that LLO significantly promoted the growth of probiotics and inhibited the inflammatory response induced by lipopolysaccharides (LPS) in RAW 264.7 cells [18]. Wu et al. investigated the effect of alginate oligosaccharides on ulcerative colitis in C57BL/6 mice [19]. The results indicate that alginate oligosaccharides suppressed pathological histological damage by inhibiting the disease activity index and histopathological scores, inhibiting colonic length shortening, and slowing down weight loss [19].

Agaro-oligosaccharides have been widely applied in the food, medicine, and cosmetic industries due to their health benefits such as anti-tumor, antioxidant, anti-inflammatory, and prebiotic effects [20]. In our previous research, the agaro-oligosaccharides prepared from the agar of *Gracilaria lemaneiformis* (AO) were prepared by HCl hydrolysis, and the anti-inflammatory effect of AO was investigated. The results show that AO remarkably inhibited LPS-induced inflammation [21]. In the current study, in order to further explore the bioactivities of AO, the effect of AO on H_2_O_2_-stimulated oxidative stress was evaluated.

## 2. Results and Discussion

### 2.1. AO Suppresses H_2_O_2_-Stimulated Oxidative Stress in Vero Cells

Seaweeds contain various bioactive compounds. In particular, seaweeds are rich in polysaccharides. The polysaccharides are the components of the seaweed cell wall. For example, alginate and fucoidan are found in brown seaweeds, and agar and carrageenan are found in red seaweeds. Agar is widely used in the food industry. Agar oligosaccharides, the hydrolysate of agar, possess various bioactivities due to their low molecular weight, good water solubility, and high absorption efficiency [22,23,24,25]. Ma et al. evaluated the anti-aging effect of the commercial pharmaceutical-grade agar oligosaccharide (CPAO) on *Drosophila melanogaster*. The results indicated that CPAO significantly prolonged the lifespan of male *Drosophila melanogaster* [26]. In addition, CPAO effectively increased the antioxidant effect of male *Drosophila melanogaster* [26]. Their further study suggests that CPAO effectively inhibited the intestinal inflammation of male *Drosophila melanogaster* by modulating the microbiota [27]. In our previous study, the anti-inflammatory effect of AO was evaluated. The results showed that AO remarkably suppressed LPS-induced inflammatory response in in vitro and in vivo models [21]. These results suggest the potential of AO in the medical, functional food, and cosmetic industries. In the current study, in order to further investigate the health benefits of AO, the in vitro and in vivo antioxidant activities of AO were evaluated.

As shown in Table 1, AO scavenged hydroxyl, alkyl, and 1,1-diphenyl-2-picrylhydrazyl (DPPH) radicals at the IC_50_ value of 1.33 ± 0.05, 4.86 ± 0.13, and 3.02 ± 0.44 mg/mL, respectively. These results indicate that AO possesses a strong free radical scavenging effect. AO showed an especially strong scavenging effect on the hydroxyl radical. Based on these results, we selected H_2_O_2_ as a stimulator to stimulate oxidative damage in the in vitro and in vivo models.

Vero cells stimulated with H_2_O_2_ were successfully used to evaluate the in vitro antioxidant activity [28]. The cytotoxicity of different concentrations of H_2_O_2_ to Vero cells was evaluated. Based on the results, we made the in vitro model and applied it to evaluate the antioxidant activity of natural products. Thus, in the present study, the in vitro antioxidant activity of AO was evaluated in H_2_O_2_-stimulated Vero cells.

**Figure 1:**
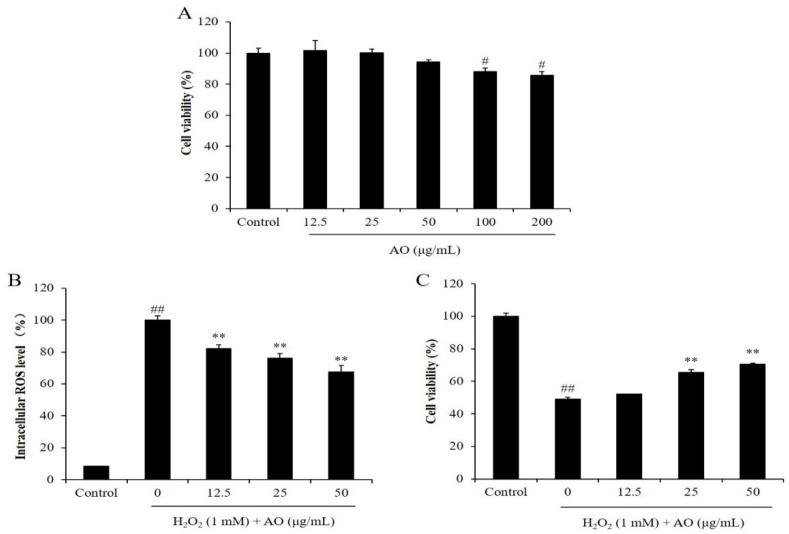
The effect of AO on H_2_O_2_-stimulated oxidative damage in Vero cells. (**A**) Cytotoxicity of AO on Vero cells; (**B**) the intracellular ROS levels of H_2_O_2_-stimulated Vero cells; (**C**) the viability of H_2_O_2_-treated Vero cells. ^#^ *p* < 0.05 and ^##^ *p* < 0.01 as compared to the control group. ** *p* < 0.01 as compared to the H_2_O_2_-treated group.

Before we investigated the in vitro antioxidant activity of AO, the cytotoxicity of AO on Vero cells was evaluated. As Figure 1A shows, the viabilities of the Vero cells treated with 12.5, 25, 50, 100, and 200 μg/mL AO were 101.50, 100.08, 94.27, 87.98, and 85.73%, respectively. According to these results, the viabilities of the Vero cells were decreased to 87.98 and 85.73% compared to the control group (100%) by treatment of 100 and 200 μg/mL, respectively. However, the viabilities of the Vero cells were all higher than 90% under the treatment of 12.5, 25, and 50 μg/mL AO (Figure 1A). These results indicated that AO showed slight cytotoxicity to Vero cells at a concentration higher than 100 μg/mL, but non-cytotoxicity under a concentration below 50 μg/mL. Based on these results, 50 μg/mL was selected as the maximum concentration of AO treatment for Vero cells in the further research.

As shown in Figure 1B, the intracellular ROS levels of the Vero cells treated with H_2_O_2_ were remarkably increased compared to the normal cells. However, the intracellular ROS levels of H_2_O_2_-treated Vero cells were decreased from 100% to 80.20, 76.06, and 67.61% by treatment of 12.5, 25, and 50 μg/mL AO, respectively (Figure 1B). These results show that AO remarkably scavenged intracellular ROS stimulated by H_2_O_2_ in a concentration-dependent manner, and suggest the potential of AO to protect cells against H_2_O_2_-stimulated damage.

**Figure 2:**
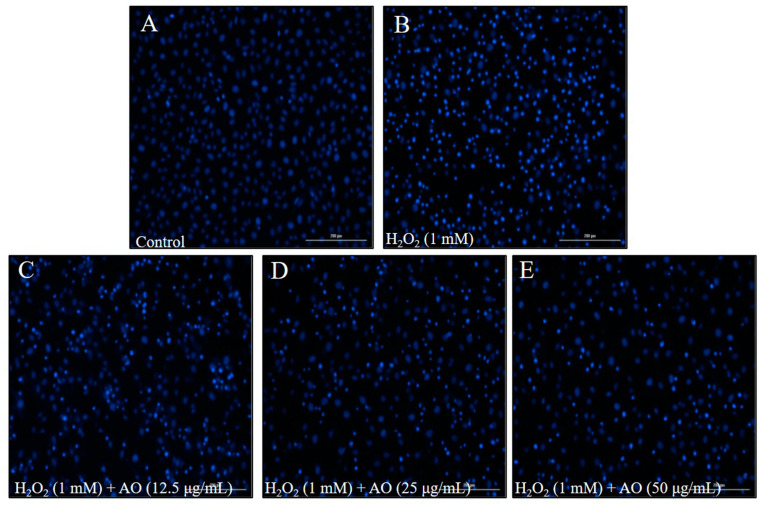
The effect of AO on H_2_O_2_-stimulated apoptosis in Vero cells. (**A**) Nuclear morphology of non H_2_O_2_-treated Vero cells; (**B**) nuclear morphology of H_2_O_2_-treated Vero cells; (**C**) nuclear morphology of Vero cells treated with 12.5 µg/mL of AO and H_2_O_2_; (**D**) nuclear morphology of cells treated with 50 µg/mL of AO and H_2_O_2_; (**E**) nuclear morphology of cells treated with 100 µg/mL of AO and H_2_O_2_.

The cytoprotective effect of AO was investigated by evaluating the viability and apoptosis level of H_2_O_2_-stimulated Vero cells. As shown in Figure 1C, the viability of H_2_O_2_-stimulated Vero cells was decreased to 48.96% from 100% (control group). However, the viabilities of Vero cells treated with 12.5, 25, and 50 μg/mL AO were increased to 52.11, 65.47, and 70.64%, respectively (Figure 1C). Furthermore, H_2_O_2_ significantly stimulated cell apoptosis; however, AO effectively protected Vero cells against H_2_O_2_-stimulated apoptosis. As Figure 2 shows, the apoptosis formation in H_2_O_2_-treated Vero cells was significantly increased. However, AO effectively suppressed H_2_O_2_-stimulated apoptosis (Figure 2). The effects show a concentration-dependent manner. The above results indicate that AO effectively and concentration-dependently alleviated H_2_O_2_-stimulated Vero cell death by suppressing apoptosis via scavenging intracellular ROS.

### 2.2. In Vivo Antioxidant Activity of AO

Zebrafish (*Danio rerio*) are a popular in vivo model used in toxicological, pharmacological, and biological research [29,30]. Zebrafish have several advantages such as easy maintenance, rapid embryonic development, and a large number of offspring. Because of these advantages, zebrafish are a popular in vivo model in toxicological and pharmaceutical research [31,32,33]. The zebrafish embryo is used as a potential model for investigating the bioactivities of natural compounds. H_2_O_2_-stimulated oxidative stress in zebrafish has been reported in previous studies [28]. Zebrafish stimulated by H_2_O_2_ were successfully used as an in vivo model to evaluate the antioxidant activities of algal polysaccharides [28,34]. Thus, in the current research, H_2_O_2_-induced zebrafish were selected as an in vivo model to evaluate the antioxidant activity of AO.

**Figure 3:**
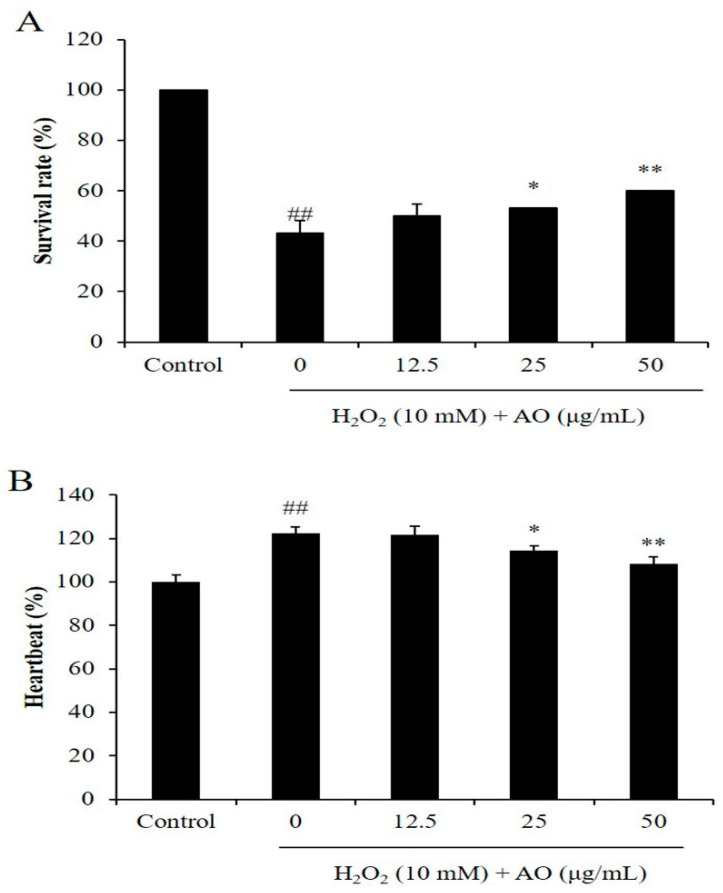
The effect of AO on H_2_O_2_-stimulated zebrafish damage. (**A**) The survival rate of H_2_O_2_-treated zebrafish; (**B**) the heartbeat of H_2_O_2_-treated zebrafish. ^##^ *p* < 0.01 as compared to the control group. * *p* < 0.05 and ** *p* < 0.01 as compared to the H_2_O_2_-treated group.

**Figure 4:**
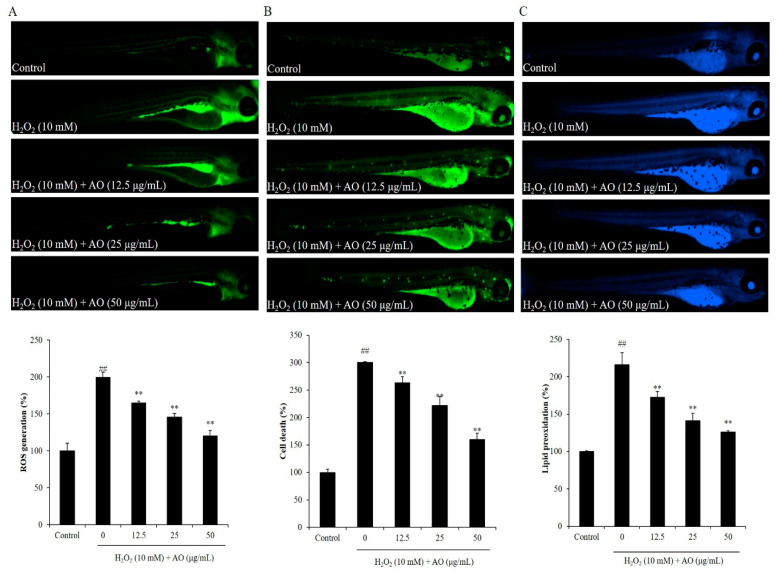
The effect of AO on H_2_O_2_-stimulated oxidative stress in zebrafish. (**A**) The protective effect of AO against H_2_O_2_-stimulated ROS production; (**B**) the protective effect of AO against H_2_O_2_-stimulated cell death; (**C**) the protective effect of AO against H_2_O_2_-stimulated lipid peroxidation. ^##^ *p* < 0.01 as compared to the control group. ** *p* < 0.01 as compared to the H_2_O_2_-treated group.

As Figure 3A shows, the survival rates of zebrafish were significantly reduced by H_2_O_2_, and improved by AO in a dose-dependent manner. H_2_O_2_ reduced the survival rate of zebrafish from 100% to 43.33% (Figure 3A). However, the survival rates of H_2_O_2_-stimulated zebrafish were increased to 50, 53.33, and 60% by treatment of 12.5, 25, and 50 μg/mL, respectively (Figure 3A). As shown in Figure 4B, H_2_O_2_ significantly stimulated heartbeat disorder, and AO effectively suppressed heartbeat disorder (Figure 3B). The heartbeat of H_2_O_2_-treated zebrafish increased to 122.51% from 100% (the control group). However, AO reduced the heartbeat from 122.51% to 121.71, 114.44, and 108.67% at the dose of 12.5, 25, and 50 μg/mL, respectively (Figure 3B). These results indicate that AO effectively increased the survival rate and suppressed heartbeat disorder in a dose-dependent manner.

The effect of AO on cell death, ROS level, and lipid peroxidation of H_2_O_2_-induced zebrafish was investigated. H_2_O_2_ stimulated ROS generation in zebrafish, and AO remarkably scavenged the ROS stimulated by H_2_O_2_ in a dose-dependent manner. As shown in Figure 4A, H_2_O_2_ significantly elevated the ROS level of zebrafish to 199.73% compared to the control group (100%). However, the ROS level of H_2_O_2_-induced zebrafish was decreased to 164.92, 145.86, and 120.43% by treatment of 12.5, 25, and 50 μg/mL AO, respectively (Figure 4A). In addition, H_2_O_2_ significantly induced cell death in zebrafish, and AO remarkably suppressed H_2_O_2_-stimulated cell death in a dose-dependent manner. The cell death of H_2_O_2_-stimulated zebrafish was increased to 300.25% from 100% (the control group), whereas the cell death of H_2_O_2_-stimulated zebrafish was decreased to 263.167, 221.68, and 159.68% via the treatment of 12.5, 25, and 50 μg/mL AO, respectively (Figure 4B). Furthermore, H_2_O_2_ stimulated lipid peroxidation in zebrafish, and AO remarkably suppressed the lipid peroxidation stimulated by H_2_O_2_ in a dose-dependent manner (Figure 4C). H_2_O_2_ elevated the lipid peroxidation of zebrafish to 216.40% compared to the control group (100%), whereas the lipid peroxidation of H_2_O_2_-stimulated zebrafish treated with a dose of 12.5, 25, and 50 μg/mL AO was decreased to 172.65, 141.21, and 126.08%, respectively (Figure 4C). These results demonstrate that AO effectively suppressed H_2_O_2_-induced in vivo oxidative damage in zebrafish via reducing ROS, cell death, and lipid peroxidation.

In summary, the above results indicate that AO remarkably protected Vero cells against H_2_O_2_-stimulated oxidative damage by scavenging intracellular ROS. In addition, AO significantly suppressed H_2_O_2_-stimulated oxidative damage by decreasing the levels of ROS, cell death, and lipid peroxidation in vivo in zebrafish.

## 3. Materials and Methods

### 3.1. Chemicals and Reagents

Fetal bovine serum (FBS), trypsin-EDTA, and penicillin-streptomycin (P/S) were purchased from Gibco-BRL (Grand Island, NY, USA). 5,5-Dimethyl-1-pyrroline N-oxide, dimethyl sulfoxide (DMSO), acridine orange, DPPH, 2,2-azobis(2-amidinopropane) hydrochloride, α-(4-pyridyl-1-oxide)-N-tert-butylnitrone, H_2_O_2_, 3-(4,5-dimethylthiazol-2-yl)-2,5-diphenyltetrazolium bromide (MTT), 1,3-bis (diphenylphosphino) propane (DPPP), and 2,7-dichlorofluorescein diacetate (DCFH_2_-DA) were purchased from Sigma-Aldrich (St. Louis, MO, USA).

### 3.2. Evaluation of Free Radical Scavenging Activity of AO

The effects of AO on scavenging hydroxyl, alkyl, and DPPH radicals were determined using an electron spin resonance spectrometer based on the protocols described by Wang et al. [35].

### 3.3. Preparation of AO

AO was prepared in our previous study [21]. In brief, agar from *Gracilaria lemaneiformis* was hydrolyzed by 0.5 M HCl at 100 °C for 3 h. The hydrolysate was filtered after cooling to room temperature. After lyophilization, the agaro-oligosaccharides were obtained and named as AO. To analyze the components of AO, AO was further purified by the Bio-Gel P2 column. The fractions were analyzed by mass spectrum. The molecular weight of the main fractions of AO were determined to be 347.0953, 653.1910, and 959.2848 m/z, respectively. This result demonstrated that the main products of AO have degrees of polymerization of 2, 4, and 6.

### 3.4. Cell Culture

Vero cells were cultured in Roswell Park Memorial Institute-1640 medium (1% P/S and 10% FBS). Vero cells were sub-cultured every 3 days. Vero cells were seeded at a concentration of 5 × 10^4^ cells/well in the 24-well plate for experiments.

### 3.5. Evaluation of the Effect of AO in H_2_O_2_-Treated Vero Cells

Before investigating the antioxidant activity of AO, the toxicity of AO on Vero cells was investigated. Vero cells were seeded for 24 h. After incubation, Vero cells were treated with AO at a concentration of 12.5, 25, 50, 100, and 200 μg/mL, respectively. After 24 h, cells were treated with MTT solution (50 μL, 2 mg/mL). The cells were incubated for 3 h. After reaction, the supernatant was removed. The formazan was dissolved in DMSO and the absorbance was measured (540 nm) using a microplate reader. To investigate the antioxidant effect of AO, the viability, levels of intracellular ROS, and apoptosis of H_2_O_2_-stimulated Vero cells were measured by MTT assay, DCF-DA assay, and Hoechst 33343 staining assay, respectively.

To measure the intracellular ROS level, Vero cells were seeded for 24 h. After incubation, the cells were treated with AO for 1 h. After incubation, the AO-treated Vero cells were treated with 1 mM H_2_O_2_. After 1 h incubation, 20 μL of 0.5 mg/mL DCFH2-DA solution was treated to each well. The cells were incubated with DCFH2-DA for 30 min. Then, the fluorescence intensity of DCF-DA was determined using a fluorescence microplate reader.

To measure the cytoprotective effect of AO, Vero cells were seeded for 24 h. Then, the Vero cells were treated with different concentrations of AO for 1 h. After incubation, the AO-treated Vero cells were treated with 1 mM H_2_O_2_. After 24 h, 50 μL of 2 mg/mL MTT solution was added to each well. The cells were incubated for 3 h. After reaction, the supernatant was removed, and the formazan was dissolved in DMSO. The absorbance was measured at 540 nm using a microplate reader.

To analyze the apoptosis level of Vero cells, Vero cells were seeded in a 24-well plate for 24 h. Then, the Vero cells were treated with different concentrations of AO for 1 h. After incubation, the Vero cells treated with AO were stimulated with 1 mM H_2_O_2_. After 6 h, the Vero cells were stained by Hoechst 33343 for 30 min. The Hoechst 33342-stained cells were observed and photographed under a fluorescence microscope.

### 3.6. Maintenance of Zebrafish

The adult zebrafish were maintained according to the previous protocol [28]. In brief, the adult zebrafish were kept in the tank under 28.5 °C and in 14/10 h light/dark cycle. The zebrafish were fed three times per day with the Tetramin flake feed supplemented with live brine shrimp. Zebrafish embryos were collected from natural spawning stimulated by the light, and the collection of embryos was completed within 30 min. 

### 3.7. Evaluation of the Effect of AO in H_2_O_2_-Treated Zebrafish

The in vivo antioxidant activity of AO was investigated using the H_2_O_2_-stimulated zebrafish model. The zebrafish embryos were treated with 12.5, 25, and 50 μg/mL AO for 1 h. After incubation, H_2_O_2_ (10 mM) was introduced into embryos, and the embryos were incubated with H_2_O_2_ until 24 h post-fertilization. The survival rates of H_2_O_2_-treated zebrafish were determined at 3 days post-fertilization [14]. The intracellular lipid peroxidation, ROS, and cell death were measured using DPPP, DCFH2-DA, and acridine orange staining, respectively [15].

### 3.8. Statistical Analysis

The data are expressed as the mean ± standard error. One-way ANOVA was used to compare the mean values of each treatment in SPSS 20.0. Significant differences between the means were identified by the Tukey test. *p* < 0.05 were considered significant.

## 4. Conclusions

In the current research, the antioxidant activities of agaro-oligosaccharides prepared from the agar of *Gracilaria lemaneiformis* (AO) were investigated using H_2_O_2_-stimulated Vero cells and zebrafish models. AO effectively alleviated H_2_O_2_-stimulated oxidative damage in in vitro and in vivo models. The present results suggest the potential of AO in the functional food and cosmetic industries. However, to broaden the application field of AO, the bioactivities of AO need further investigation.

## Figures and Tables

**Table 1:** The free radical scavenging effect of AO.

Free Radical	IC_50_ Values (mg/mL)
DPPH	3.02 ± 0.44
Alkyl	4.86 ± 0.13
Hydroxyl	1.33 ± 0.05

## Data Availability

Not applicable.
